# Sex and Atrial Fibrillation Independently Stratify Cardiac Remodeling and Outcomes in Heart Failure with Preserved Ejection Fraction

**DOI:** 10.3390/biomedicines14051160

**Published:** 2026-05-20

**Authors:** Diana-Ruxandra Hădăreanu, Flavia-Mihaela Stoiculescu, Călin-Dinu Hădăreanu, Maria-Livia Iovănescu, Anca Mihu-Marinescu, Georgică-Costinel Târtea, Ionuț Donoiu, Oana Munteanu-Mirea, Răzvan-Ilie Radu, Eugen-Nicolae Țieranu, Octavian Istrătoaie, Cristina Florescu

**Affiliations:** 1 Department of Cardiology, University of Medicine and Pharmacy of Craiova, 2 Petru Rares St., 200349 Craiova, Romaniageorgica.tartea@umfcv.ro (G.-C.T.); ionut.donoiu@umfcv.ro (I.D.); oana.munteanu@umfcv.ro (O.M.-M.);; 2 Doctoral School, University of Medicine and Pharmacy of Craiova, 2 Petru Rares St., 200349 Craiova, Romania; 3 Department of Cardiology, Clinical Emergency County Hospital of Craiova, 1 Tabaci St., 200642 Craiova, Romania; 4 Department of Cardiovascular Surgery, Clinical Emergency County Hospital of Craiova, 1 Tabaci St., 200642 Craiova, Romania; 5 Department of Cardiology, Filantropia Clinical Hospital, 28 Sararilor St., 200516 Craiova, Romania; 6 Department of Cardiology, Carol Davila University of Medicine and Pharmacy, 8 Eroii Sanitari Bld., 050474 Bucharest, Romania; razvan-ilie.radu@umfcd.ro; 7 Department of Interventional Cardiology, Prof. Dr. C. C. Iliescu Emergency Institute for Cardiovascular Diseases, 258 Fundeni St., 022328 Bucharest, Romania

**Keywords:** heart failure with preserved ejection fraction, atrial fibrillation, sex differences, cardiac remodeling, prognosis, risk stratification

## Abstract

**Background/Objectives**: Atrial fibrillation (AF) is common in heart failure with preserved ejection fraction (HFpEF) and is associated with worse symptoms and prognosis. Emerging evidence suggests that sex modifies the AF–HFpEF relationship through differences in atrial remodeling, comorbidity burden, and hemodynamic vulnerability. This study aimed to evaluate how sex and AF jointly relate to differences in cardiac structure, clinical characteristics, and outcomes in HFpEF. **Methods**: We retrospectively analyzed 622 patients with HFpEF admitted between January 2019 and May 2023. Patients were categorized into four predefined clinical subgroups: women without AF, women with AF, men without AF, and men with AF. The primary endpoint was first rehospitalization for HF decompensation. **Results**: Over a mean follow-up of 48.6 ± 16.4 months, 181 patients (29.1%) were rehospitalized for worsening HF, with the highest event burden observed in men with AF. Sex and AF were each associated with distinct clinical and remodeling profiles, without significant sex-by-AF interaction effects. AF was independently associated with a higher risk of HF rehospitalization (HR 1.45, 95% CI 1.06–1.99, *p* = 0.021), whereas female sex was protective (HR 0.71, 95% CI 0.53–0.97, *p* = 0.032). Men with AF exhibited the most adverse remodeling profile, characterized by the largest unindexed left atrial and left ventricular dimensions, the highest prevalence of significant tricuspid regurgitation, and the lowest event-free survival (HR 1.92, 95% CI 1.23–2.99, *p* = 0.004). In contrast, women with AF more frequently displayed concentric remodeling and significant mitral regurgitation. Independent predictors of rehospitalization included higher NYHA functional class and lower left ventricular EF within the preserved EF range. **Conclusions**: Sex and AF were independently associated with substantial differences in cardiac structure, clinical characteristics and prognosis in HFpEF. Men with AF represent the highest-risk subgroup, driven by more advanced structural remodeling and valvular dysfunction. These findings suggest that simple sex- and rhythm-based classification may provide complementary information for risk stratification and management in HFpEF. Further validation in independent cohorts is warranted.

## 1. Introduction

Atrial fibrillation (AF) and heart failure (HF) with preserved ejection fraction (HFpEF) frequently coexist and share a complex, bidirectional relationship driven by overlapping risk factors, atrial structural remodeling, and hemodynamic deterioration [1,2]. AF is present in more than one-third of patients with newly diagnosed HF and in up to half of those with chronic HFpEF, substantially increasing morbidity and mortality [1,2,3]. This association is particularly relevant in women, who exhibit a higher symptom burden, greater prevalence of persistent AF, and increased risk of HF-related complications [4,5]. Persistent or permanent AF, in particular, is strongly associated with subsequent HF development, especially in women, suggesting underlying biological susceptibilities such as enhanced atrial structural remodeling.

The central pathophysiological substrate linking AF and HF is atrial cardiomyopathy—an integrated spectrum of structural, architectural, contractile, and electrophysiological alterations of atrial tissue [6]. Histological studies have demonstrated enhanced atrial fibrosis, impaired myocardial compliance, capillary rarefaction, fibroblast proliferation, and cardiomyocyte hypertrophy in patients with AF and/or HF [7]. Notably, female sex and HF are major determinants of a predominantly fibrotic atrial cardiomyopathy phenotype, while men more frequently exhibit hypertrophic remodeling [7]. From a hemodynamic perspective, AF—irrespective of ventricular rate—impairs atrial reservoir and booster pump function, leading to increased left atrial pressures, loss of atrial systole, irregular ventricular filling, functional mitral regurgitation, and ultimately pulmonary congestion [2]. HF further increases AF susceptibility through elevated atrial afterload, neurohormonal activation, systemic inflammation, and progressive left atrial structural remodeling [1]. Moreover, adherence to the ABC pathway (A—Avoid stroke; B—Better symptom control; C—Comorbidity and cardiovascular risk management) combined with optimal HF medical therapy has been associated with improved clinical outcomes [8,9].

Given the prognostic relevance of AF–HFpEF coexistence, improved understanding of sex-specific remodeling patterns, clinical characteristics, and outcomes is essential to inform tailored management strategies. Accordingly, the aim of this study was to systematically evaluate how sex and AF are associated with differences in the clinical presentation and prognosis of patients with HFpEF. Specifically, we sought to: (i) characterize sex-specific structural and functional cardiac changes—including the prevalence of atrioventricular valve regurgitation—and compare the clinical characteristics of four predefined HFpEF subgroups based on sex and the presence or absence of AF; and (ii) identify variables independently associated with HF rehospitalization in HFpEF. Although AF and sex differences have each been individually associated with HFpEF outcomes, less in known regarding whether combining these two routinely available variables may improve risk stratification in everyday clinical care.

## 2. Materials and Methods

### 2.1. Study Population and Data Collection

This retrospective observational study was conducted at the Clinical Emergency County Hospital of Craiova (Romania), as part of the REMO-FIB (“The assessment of cardiac remodeling by advanced imaging techniques in patients with atrial fibrillation”) research project. All admissions registered between 1 January 2019 and 31 May 2023 were screened using the institutional electronic medical record. During this interval, a total of 15,160 hospitalizations were examined. Patients were eligible if they fulfilled contemporary European Society of Cardiology diagnostic criteria for HFpEF, requiring typical symptoms and signs of HF, a left ventricular (LV) EF ≥ 50%, and structural or functional evidence of diastolic dysfunction (presence of concentric LV remodeling/hypertrophy, left atrial enlargement, indices of elevated LV filling pressures—E/e’ ratio, elevated serum natriuretic peptides). Admissions related to acute coronary syndromes, acute pulmonary embolism, resuscitated cardiac arrest, advanced atrioventricular block, sustained ventricular arrhythmias, or other acute conditions that could confound HF assessment were excluded. Patients with missing key clinical, echocardiographic, or laboratory information, as well as those lacking at least 1 year of follow-up, were also removed. After careful verification, a final cohort of 622 HFpEF patients with complete data and outcome ascertainment was retained for analysis. The study was conducted in accordance with the Declaration of Helsinki and was approved by the Ethics Committee of the University of Medicine and Pharmacy of Craiova, Romania (approval number 117; date of approval: 10 February 2025).

Data were extracted from the hospital’s electronic medical records using a standardized data collection form. Demographic characteristics, cardiovascular risk factors, comorbidities, New York Heart Association (NYHA) functional class, and chronic medical therapies were extracted from the index hospitalization. Laboratory variables included hemoglobin, serum sodium, glucose, creatinine, aminotransferases, NT-proBNP, and renal function estimated using the CKD-EPI equation. Because the primary aim of the present study was to evaluate the combined effects of sex and AF in HFpEF, patients were categorized into four predefined clinical subgroups: (1) women without AF, (2) women with AF, (3) men without AF, and (4) men with AF. AF status was ascertained from index hospitalization ECGs in conjunction with available longitudinal clinical documentation, including prior ECG or rhythm-monitoring records, discharge summaries, and cardiology follow-up records. When available, AF subtype was classified according to temporal-pattern classification as paroxysmal, persistent, or permanent, since the 2024 ESC guidelines no longer use valvular/non-valvular AF as a classification framework [9]. Paroxysmal AF was defined as self-terminating AF with documented sinus rhythm between episodes, while persistent and permanent AF were defined according to standard clinical documentation [9]. AF subtype information was retrieved for descriptive purposes only and was not used for subgroup analyses due to a marked imbalance between subtypes. This approach allowed for characterization of sex-specific arrhythmic clinical groups relevant to HFpEF physiology and prognosis.

All patients underwent comprehensive transthoracic echocardiographic evaluation following standardized acquisition and measurement protocols recommended by the European Association of Cardiovascular Imaging [10]. Quantitative parameters included interventricular septal thickness, posterior wall thickness, LV end-diastolic diameter, LV mass (calculated using the Devereux formula), relative wall thickness, and LVEF by the biplane Simpson method [11]. Left atrial anteroposterior diameter, tricuspid annular plane systolic excursion (TAPSE), Doppler-derived systolic pulmonary artery pressure, and multiparametric assessment of mitral and tricuspid regurgitation were also recorded.

### 2.2. Follow-Up and Outcome Definition

Patients were followed for the occurrence of a first HF rehospitalization, defined as an unplanned admission requiring intravenous diuretics, vasodilators, or inotropic support. Events were confirmed using hospital documentation, outpatient records, and telephone contact. Individuals without an event were censored at the last known clinical encounter.

### 2.3. Statistical Analysis

Statistical analyses were performed using IBM SPSS Statistics, version 23 (IBM Corp., Armonk, NY, USA). Continuous variables were tested for normality using the Shapiro–Wilk test and reported as mean ± standard deviation. Because NT-proBNP showed a markedly skewed distribution, it was logarithmically transformed before regression analysis and analyzed as a log-transformed continuous variable in the univariable Cox model. Intergroup comparisons were conducted using one-way ANOVA; additionally, when evaluating the combined effects of sex and AF, two-way ANOVA models were constructed including sex, AF, and their interaction term (sex × AF), with homogeneity of variances assessed by Levene’s test. A significant interaction term was interpreted as indicating that the association of atrial fibrillation with the studied variable differed between women and men. Post hoc analyses were performed using Tukey’s HSD in the presence of equal variances and the Games–Howell procedure otherwise. Categorical variables were expressed as counts and percentages and compared using the Pearson’s chi-square test. Adjusted standardized residuals were inspected to identify cells contributing most to significant associations, with thresholds of ±1.96, ±2.58, and ±3.29 indicating significance at *p* < 0.05, *p* < 0.01, and *p* < 0.001, respectively. Cox proportional hazards regression was applied to determine variables associated with HF rehospitalization. Univariable analyses were followed by a multivariable model including variables selected a priori based on clinical plausibility and known prognostic relevance in patients with HFpEF. Harrell’s C-index was calculated for baseline and extended models. Additional multivariable Cox models including a formal sex × AF interaction term were constructed. A four-group sex/rhythm model was also evaluated to assess subgroup-specific risk. Time-to-event analyses were performed using Kaplan–Meier estimates and compared using the log-rank test. A two-sided *p*-value < 0.05 was considered statistically significant.

## 3. Results

### 3.1. Baseline Characteristics of the Entire Study Population and Across the Four Sex–Atrial Fibrillation Subgroups

A total of 622 patients with HFpEF were included. The four predefined AF–sex subgroups showed consistent and clinically meaningful differences at baseline (Table 1). Among patients with AF, AF was classified as paroxysmal in 5 patients (1.8%), persistent in 53 patients (18.8%), and permanent in 224 patients (79.4%). Because paroxysmal AF was rare and sustained AF phenotypes predominated, no subtype-specific comparative analyses were performed.

#### 3.1.1. Demographics and Laboratory Findings

Age differed significantly across the four groups (ANOVA *p* < 0.001), with women with AF representing the oldest subgroup. Serum creatinine (*p* = 0.005) and estimated glomerular filtration rate (*p* = 0.003) also varied, suggesting a modestly greater renal burden in AF subgroups. Hemoglobin also differed significantly across the four subgroups (ANOVA *p* < 0.001). Other laboratory parameters—including electrolytes, liver enzymes, glucose, and NT-proBNP—did not show statistically significant between-group differences.

#### 3.1.2. Cardiac Structure and Function

Differences in unindexed structural echocardiographic parameters were evident across subgroups. LV end-diastolic diameter, LV mass, and left atrial diameter differed significantly across groups (all *p* < 0.001). Post hoc analysis showed that both AF groups exhibited significantly larger left atrial size and higher LV mass compared with patients without AF, irrespective of sex. LVEF also differed modestly across the four subgroups (*p* = 0.039). Post hoc comparison indicated that women with AF had slightly lower LVEF than women without AF (*p* = 0.048), although all values remained within the range consistent with the HFpEF definition. Relative wall thickness differed between groups (*p* = 0.025), reflecting variation in concentric remodeling, while right-sided parameters such as TAPSE and systolic pulmonary artery pressure did not reach statistical significance.

To formally assess whether the associations between AF and clinical, laboratory, or echocardiographic characteristics differed by sex, two-way ANOVA models including sex, AF, and their interaction term were performed. No significant sex × AF interactions were observed for any of the analyzed variables, indicating that the associations of AF with these parameters were consistent in women and men.

#### 3.1.3. Clinical Characteristics and Comorbidities

Significant between-group differences were observed in both mitral and tricuspid regurgitation severity distributions. Adjusted residuals showed that women with AF were overrepresented among those with significant (more than mild) mitral regurgitation (*p* < 0.001), whereas men with AF were more frequently affected by significant (more than mild) tricuspid regurgitation (*p* = 0.007). Hypertension, hypercholesterolemia, smoking status, and severity of HF signs and symptoms were unevenly distributed across the groups (all *p* ≤ 0.001), with the highest clustering of adverse comorbidities and greater clinical severity at index hospitalization observed in AF subgroups. Diabetes and chronic kidney disease showed no statistically significant between-group differences.

#### 3.1.4. Pharmacological Therapies

Marked between-group differences in medical therapy were observed. Use of loop diuretics (*p* < 0.001), angiotensin converting enzyme inhibitors or angiotensin receptor neprilysin inhibitors (*p* = 0.001), mineralocorticoid receptor antagonists (*p* < 0.001), β-blockers (*p* = 0.017), and antiarrhythmics (*p* < 0.001) varied significantly. Adjusted residuals indicated more frequent use of diuretics and mineralocorticoid receptor antagonists among AF patients, consistent with a greater degree of congestion and disease severity. In contrast, use of sodium–glucose cotransporter-2 inhibitors was similar across the four subgroups.

#### 3.1.5. Clinical Outcomes

Rehospitalization for worsening HF differed significantly between the groups (*p* = 0.003), with higher rates observed in AF subgroups. During a mean follow-up of 49 ± 16 months, 181 patients (29.1%) experienced at least one HF rehospitalization, with the greatest event burden observed in men with AF, followed by women with AF, whereas both non-AF groups showed substantially lower rates. In contrast, all-cause mortality alone did not differ significantly (*p* = 0.907), although absolute numbers were low.

### 3.2. Cox Regression Analysis

At univariable analysis, multiple clinical and echocardiographic factors were associated with the risk of HF rehospitalization (Table 2). Female sex (HR 0.705, 95% CI 0.526–0.945, *p* = 0.019) was associated with a lower risk of HF rehospitalization. Conversely, AF was associated with a higher risk of HF rehospitalization (HR 1.490, 95% CI 1.112–1.998, *p* = 0.008). Functional and structural variables showed consistent associations with outcomes: higher NYHA class, lower LVEF, larger left atrial diameter, elevated systolic pulmonary artery pressure, and reduced TAPSE were each associated with a greater likelihood of events. Moderate-to-severe mitral regurgitation and tricuspid regurgitation were also significantly associated with HF rehospitalization in univariable analysis.

In the multivariable model, several variables retained independent prognostic significance (Table 3). Age showed a modest inverse association with rehospitalization risk in both univariable (HR 0.985, 95% CI 0.973–0.997, *p* = 0.017) and multivariable (HR 0.981, 95% CI 0.969–0.994, *p* = 0.003) analyses. Female sex remained independently associated with a lower risk of HF rehospitalization, while AF remained independently associated with a higher risk of rehospitalization (HR 1.449, *p* = 0.021). Furthermore, NYHA class and LVEF both remained strongly associated with outcomes. Finally, while sodium–glucose cotransporter 2 inhibitors (SGLT2is) use was associated with rehospitalization risk in univariable analysis, this association did not remain statistically significant in the multivariable model.

In a sensitivity analysis, AF remained independently associated with HF rehospitalization (HR 1.52, 95% CI 1.10–2.10, *p* = 0.010). Findings were therefore consistent with the primary analysis.

### 3.3. Additional Prognostic Analyses

In a parsimonious multivariable Cox model (Table 4) including age, sex, AF, NYHA class, and LVEF, AF remained independently associated with HF rehospitalization (HR 1.51, 95% CI 1.11–2.06, *p* = 0.008), while female sex remained independently associated with lower risk (HR 0.73, 95% CI 0.54–0.99, *p* = 0.042). In an expanded model (Table 5) additionally including estimated glomerular filtration rate, SGLT2 inhibitor use, mineralocorticoid receptor antagonist use, loop diuretic use, and LV end-diastolic diameter, AF remained independently associated with rehospitalization (HR 1.45, 95% CI 1.05–1.98, *p* = 0.023). No significant sex × AF interaction was identified (likelihood-ratio *p* = 0.128), indicating additive rather than synergistic prognostic associations. Addition of sex and AF to a baseline model including age, NYHA class, and LVEF modestly improved discrimination for HF rehospitalization (Harrell’s C-index 0.603 vs. 0.622). In the adjusted four-group model (Table 6), men with AF had the highest risk of rehospitalization (HR 1.85, 95% CI 1.18–2.89, *p* = 0.007).

### 3.4. Kaplan–Meier Survival Analysis

**Figure 1:**
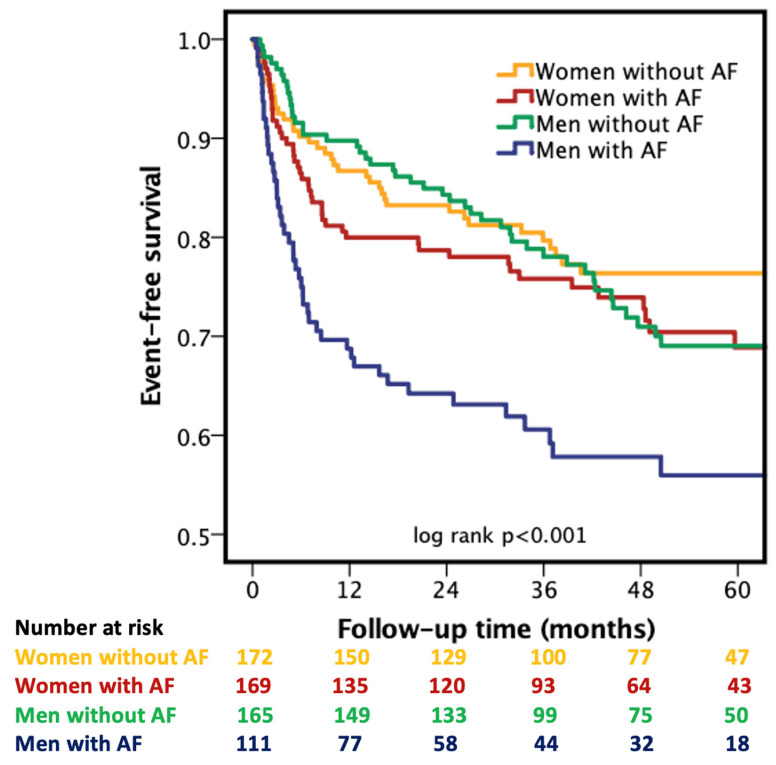
Kaplan–Meier Curves Showing the Sex-Specific Influence of Atrial Fibrillation on Event-Free Survival in HFpEF. Kaplan–Meier curves were compared using the log-rank test.

Event-free survival differed significantly across the four AF–sex subgroups (log-rank *p* < 0.001, Figure 1). The most pronounced separation was observed in men with AF, who showed an early and sustained increase in rehospitalization risk over the course of follow-up. In contrast, women with AF demonstrated an intermediate trajectory, with event-free survival largely overlapping with both non-AF groups after the first year of follow-up. Men and women without AF had the most favorable and largely comparable outcomes. These findings indicate that the excess risk associated with AF in HFpEF is primarily driven by male patients, supporting a sex-specific vulnerability profile.

## 4. Discussion

### 4.1. Summary of Key Findings

In this real-world cohort of 622 patients with HFpEF stratified by sex and AF status, we found that: (1) AF was independently associated with a higher risk of HF rehospitalization; (2) female sex was independently associated with a lower risk of HF rehospitalization, even after adjustment for clinical and echocardiographic variables; (3) men with AF represented the highest-risk subgroup, with the steepest decline in event-free survival; (4) structural echocardiographic characteristics and valvular involvement differed substantially between groups, with more significant mitral regurgitation in women with AF and more significant tricuspid regurgitation in men with AF; and (5) NYHA class and LVEF emerged as key, guideline-consistent variables independently associated with outcomes.

Our results align with current European Society of Cardiology guidelines on AF and HF, which recognize AF as a central determinant of symptoms and prognosis in HFpEF, rather than as a simple comorbidity [3,9]. Furthermore, by stratifying patients according to sex and cardiac rhythm into four easily identifiable clinical categories, our study refines this perspective and reveals a particularly high-risk profile in men with AF. No significant sex-by-AF interaction was observed, suggesting additive effects. Taken together, these findings indicate that combining sex and AF status may help identify clinically relevant differences in HFpEF presentation and prognosis, although the incremental prognostic improvement observed in our study was modest.

### 4.2. Sex–Atrial Fibrillation Interaction and Atrial Cardiomyopathy

Notably, AF in our cohort was almost exclusively sustained, with the vast majority of patients exhibiting persistent or permanent AF and only rare cases of paroxysmal AF. Accordingly, the observed sex-specific remodeling patterns and rehospitalization risk primarily reflect advanced atrial cardiomyopathy associated with sustained AF phenotypes. Extrapolation to paroxysmal AF should therefore be made cautiously. This mirrors the observations from large epidemiologic studies [4,12] which showed that AF markedly increases the risk of incident HF and mortality, with a stronger relative impact in women but a higher absolute burden of events in men.

At the same time, our finding that female sex was independently associated with a lower risk of HF rehospitalization in HFpEF contrasts with the higher stroke and mortality risk often reported in women with AF in population-based cohorts [13,14]. Women with new-onset AF had a higher risk of stroke than men despite similar treatment, especially at older ages, suggesting that thromboembolic vulnerability may be greater in women, while HF decompensation risk is more strongly modulated by structural and hemodynamic factors in males [14]. Women typically develop AF later and more often present with HFpEF, whereas men more frequently exhibit chamber dilation and volume overload [15]. Our HFpEF cohort is consistent with this pattern: women with AF were older and more often displayed concentric remodeling, while men with AF had larger unindexed LV dimensions and mass. In a pooled individual-patient analysis of CHARM-Preserved, I-Preserve, and TOPCAT, Dewan et al. reported that women with HFpEF were older, more frequently hypertensive or obese, and had worse NYHA class and quality-of-life scores, yet exhibited smaller LV volumes and higher LVEF than men. Importantly, despite a greater symptom burden, women had similar heart-failure hospitalization rates but a significantly lower risk of cardiovascular and sudden death than men [16].

Conversely, the concept of atrial cardiomyopathy offers a useful framework for interpreting the structural differences observed in our cohort [6]. The CATCH ME study histologic analysis [7] showed that women and patients with HF and persistent AF more commonly exhibit a fibrotic atrial phenotype, characterized by increased endomysial fibrosis and extracellular matrix expansion. In contrast, men tend to display a hypertrophic form of atrial cardiomyopathy, marked by cardiomyocyte enlargement and chamber dilation. The structural differences identified in our cohort are consistent with the patterns observed in the aforementioned study.

Beyond echocardiographic and histologic characterization, invasive electroanatomic mapping studies provide complementary mechanistic insights into sex-specific atrial remodeling. Recent data have shown that women exhibit a faster and more pronounced progression of adverse left atrial substrate remodeling, characterized by a higher burden of low-voltage areas and atrial fibrosis, despite smaller left atrial chamber dimensions [17]. This predominantly fibrotic atrial cardiomyopathy phenotype has been associated with increased atrial stiffness and impaired reservoir function, which may contribute to elevated filling pressures and functional mitral regurgitation in women with HFpEF [17].

Although both AF subgroups exhibited structural remodeling, the patterns differed between sexes. Women with AF exhibited smaller unindexed ventricular cavities, more concentric remodeling, and a higher prevalence of mitral regurgitation. In contrast, men showed larger unindexed chamber dimensions and more frequent tricuspid regurgitation. Consistent with our findings, comprehensive imaging studies demonstrate that AF promotes remodeling across all four cardiac chambers [18], and experimental studies [19] show that AF drives oxidative stress, abnormal calcium handling, and structural remodeling, whereas HFpEF adds chronic pressure overload and systemic inflammation. The combined effect is a continuum of atrial cardiomyopathy whose manifestations differ substantially between men and women.

Importantly, formal interaction testing using two-way ANOVA did not demonstrate a statistically significant sex × AF interaction for clinical, laboratory, or echocardiographic variables. These findings support an additive, rather than synergistic, effect of sex and AF on HFpEF disease expression. Risk stratification may still be improved by combining independent predictors, as reflected by the improved C-index and the particularly adverse prognosis observed in men with AF.

### 4.3. Why Men with Atrial Fibrillation Experience the Poorest Outcomes

Kaplan–Meier curves in our study showed the steepest and earliest separation for men with AF, who consistently exhibited the highest rates of HF rehospitalization, while women with AF followed an intermediate course and both non-AF groups had better and largely overlapping survival trajectories. Additional support for a sex-specific susceptibility to AF-related decompensation is provided by real-world short-term outcome data. In a Korean cohort of older adults with HF, coexisting AF markedly increased 90-day readmission rates in both sexes, but the magnitude of risk was substantially higher in men [20].

Several factors are likely to contribute. First, more advanced geometric remodeling, a well-recognized marker of disease severity and adverse prognosis in both HF and AF [1,19]. Second, the higher burden of tricuspid regurgitation is clinically important. The higher prevalence of tricuspid regurgitation in men with AF likely contributed to worse outcomes. AF-related right atrial enlargement and annular dilation are well-known drivers of functional tricuspid regurgitation [21], since AF contributes not only to left-sided but also right-sided impairment [22]. Moderate-to-severe tricuspid regurgitation is independently associated with HF hospitalization and mortality in AF [23], and recent evidence demonstrates that tricuspid regurgitation in HFpEF is frequently related to right atrial remodeling, forming a type of atrial functional tricuspid regurgitation that carries adverse prognostic implications [24,25]. Our study also revealed moderate and severe tricuspid regurgitation to be associated with a higher risk of rehospitalization. Third, interactions with comorbidities and neurohormonal activation play a key role. Testosterone, higher sympathetic tone, and greater prevalence of obesity, sleep-disordered breathing, and metabolic risk factors in men may augment AF-related hemodynamic stress and promote decompensation, particularly in HFpEF [15]. Finally, while population-level analyses [4,12,14], highlight a relatively stronger mortality and stroke risk in women with AF, our HFpEF-specific cohort suggests that AF amplifies HF rehospitalization risk to a greater extent in men. This difference likely reflects the HFpEF phenotype and the dominance of congestion-driven events captured by our endpoint, rather than thromboembolic events.

Notably, in the broader HF population, sex alone has not consistently predicted adverse outcomes. In the REAL-HF registry, female sex was not independently associated with short- or long-term rehospitalization or mortality [24]. In contrast, within our HFpEF-specific cohort, female sex remained independently associated with a lower risk of HF rehospitalization, suggesting disease-specific mechanisms rather than a universal sex-related prognostic effect. From a clinical perspective, men with AF represented the subgroup with the highest observed rehospitalization risk in our cohort, and may warrant closer clinical surveillance and optimization of HF management strategies.

### 4.4. Predictors of Rehospitalization

In the multivariable Cox analysis, AF, more advanced NYHA class, and lower LVEF within the preserved EF range were independently associated with HF rehospitalization, while female sex was independently associated with a lower risk of HF rehospitalization. These findings align with established HFpEF prognostic markers [3]. Even modest reductions in LVEF may indicate impaired contractile reserve [19], and significant mitral regurgitation likely reflects advanced remodeling with AF-related annular dilation. Our group previously developed the AD_2_NNER score to estimate the risk of rehospitalization in HFpEF and HF with mildly reduced EF, showing that integrating simple clinical and echocardiographic variables yields robust prognostic information. The present results are consistent with this framework, as AF, mitral regurgitation severity, NYHA class, and LVEF were also key variables associated with outcome [25].

SGLT2is use was associated with rehospitalization in univariable analysis but did not remain statistically significant in the multivariable model. This finding should therefore be interpreted with caution and should not be interpreted as evidence of harm. Given the retrospective nature of the study and the time period of patient inclusion (2019–2023), SGLT2is use in our cohort was primarily related to the presence of type 2 diabetes mellitus rather than systematic prescription as HF-directed therapy in HFpEF. The landmark DELIVER [26] and EMPEROR-Preserved [27] trials, which established SGLT2 inhibitors as foundational therapy for HFpEF, were published in 2022, and guideline incorporation occurred subsequently [28]. Accordingly, the univariable association most likely reflects confounding by indication, as patients receiving SGLT2is may have had a greater cardiometabolic comorbidity burden and a higher baseline risk of HF rehospitalization. Contemporary evidence demonstrates that SGLT2 inhibitors significantly reduce HF hospitalizations and improve quality of life in HFpEF across the ejection fraction spectrum, and current guidelines recommend their use in all patients with HFpEF lacking contraindications [28].

Beyond structural and hemodynamic markers, our data should be interpreted in light of evolving evidence that supports comprehensive management strategies for AF–HF populations. The risk factor modification trials (LEGACY, CARDIO-FIT, REVERSE-AF) [29] showed that large weight loss and fitness gains reduce AF burden and promote reverse remodeling in both sexes, with fitness improvement conferring particularly pronounced benefit in women. Furthermore, patients with AF and HF who received both guideline-directed HF therapy and management consistent with the ABC pathway had lower mortality than those managed less intensively, underscoring the importance of integrating HF optimal medical therapy, anticoagulation, symptom control, and comorbidity management [8].

Regarding rhythm versus rate control strategies, emerging evidence suggests potential benefits of rhythm control in HFpEF with AF. The EAST-AFNET 4 trial demonstrated that early rhythm control (within 1 year of AF diagnosis) reduced cardiovascular death, stroke, and HF hospitalizations compared to rate control, with benefits observed across the HF spectrum including HFpEF [30,31]. In a Get With the Guidelines Heart Failure registry analysis specifically focused on HFpEF patients with AF, rhythm control was associated with lower 1-year all-cause mortality compared to rate control [32]. While catheter ablation has shown particularly robust benefits in HF patients with AF, the optimal strategy in HFpEF requires individualized decision-making considering symptom burden, comorbidities, and patient preferences [33]. Taken together, these data suggest that patients in our highest-risk subgroup—men with AF, marked remodeling, and significant tricuspid regurgitation—might particularly benefit from aggressive risk factor modification, structured integrated care, early consideration of rhythm control where feasible, and optimization of guideline-directed medical therapy including SGLT2 inhibitors. Importantly, these predictors are readily available in routine clinical practice, reinforcing the feasibility of individualized risk stratification in HFpEF.

### 4.5. Clinical Implications

From a practical perspective, these findings suggest that combining sex and AF status may offer additional clinical value when evaluating risk in HFpEF. In our cohort, men with AF represented a particularly high-risk subgroup, whereas women without AF have a comparatively more favorable prognosis. 

The utility of simple individualized strategies in HFpEF is further supported by our prior observation that sex–diabetes classification identifies a similar gradient of risk, with diabetic men representing the highest-risk subgroup and women without diabetes showing the most favorable prognosis [34]. Together, these findings suggest that combining readily available clinical variables—sex, rhythm status, and metabolic comorbidities—can meaningfully stratify HFpEF patients for personalized management.

Second, the distinct distribution of mitral and tricuspid regurgitation across these subgroups, together with the independent prognostic value of significant mitral regurgitation and the emerging recognition of atrial functional tricuspid regurgitation as a marker of biatrial myopathy in HFpEF, underscores the importance of routinely evaluating and monitoring both mitral and tricuspid regurgitation in HFpEF, particularly in patients with AF [35]. Third, because AF is independently associated with rehospitalization, its presence in HFpEF should prompt proactive management, including careful volume assessment, optimization of guideline-directed HF therapy (particularly SGLT2 inhibitors), individualized rhythm or rate control strategies with consideration of early rhythm control in appropriate candidates, and targeted risk factor modification [3,29]. Finally, our data suggest that women and men may benefit from different emphases in management. In women, closer attention to fibrotic remodeling, mitral regurgitation severity, blood pressure control, and metabolic risk factors may be particularly relevant [14,15]. In men, more assertive treatment of volume overload, tricuspid regurgitation, and sleep-disordered breathing, as well as timely consideration of rhythm control, may be especially important.

### 4.6. Limitations

This was a single-center retrospective study, which may limit generalizability. AF subtype was highly unbalanced, with the vast majority of patients exhibiting sustained AF (persistent or permanent); therefore, findings primarily reflect the prognostic implications of sustained AF in HFpEF. Limited representation of paroxysmal AF precluded adequately powered subtype-specific analyses. Left atrial remodeling was assessed using anteroposterior diameter rather than volume index due to inconsistent availability, and echocardiographic chamber dimensions were analyzed primarily as absolute rather than body-size indexed because complete anthropometric data were not available. Advanced atrial parameters such as left atrial strain were also not consistently available. Residual confounding cannot be excluded as well, since important HFpEF modifiers such as body mass index (and presence of obesity), frailty, and sleep-disordered breathing data were not consistently available and could not be included in the analysis. The study period (2019–2023) preceded widespread implementation of SGLT2 inhibitors as guideline-directed HFpEF therapy. In addition, this period overlapped with the COVID-19 pandemic, and although the primary endpoint was HF rehospitalization, residual confounding related to SARS-CoV-2 infection cannot be excluded as well [36]. Finally, the endpoint focused on HF rehospitalization; stroke and cardiovascular mortality were infrequent, limiting comparability with AF cohorts in which thromboembolic outcomes predominate.

## 5. Conclusions

In this large real-world HFpEF cohort, sex and AF were independently associated with differences in cardiac structure and clinical presentation. Men with AF represented the subgroup with the most advanced remodeling, characterized by the largest cardiac chambers in absolute size, highest burden of tricuspid regurgitation, and highest risk of HF rehospitalization, whereas women—particularly those in sinus rhythm—demonstrated more favorable clinical outcomes. Sex and AF offer complementary prognostic information and may support simple bedside risk stratification in HFpEF. Our findings should be interpreted within HFpEF patients with sustained AF rather than generalized to all AF phenotypes. Future prospective studies should evaluate whether sex- and rhythm-tailored therapeutic approaches—including early rhythm control in high-risk men with AF, targeted management of tricuspid regurgitation, and optimization of guideline-directed medical therapy including SGLT2 inhibitors—can improve outcomes in these distinct HFpEF populations.

## Figures and Tables

**Table 1:** Baseline Demographic, Laboratory, and Echocardiographic Measures Across Sex–Atrial Fibrillation Subgroups in HFpEF.

Variable	All Patients (N = 622)	Non-AF Women (G1, n = 174)	AF Women (G2, n = 170)	Non-AF Men (G3, n = 166)	AF Men (G4, n = 112)	ANOVA *p*-Value
Age (years)	74 ± 11 (23–102)	74 ± 11 (45–93)	76 ± 9 (36–102)	70 ± 12 (23–94)	74 ± 9 (52–95)	<0.001 ^1^
NT-proBNP (pg/mL)	4649 ± 6889	4360 ± 7341	5476 ± 5481	3309 ± 5876	5410 ± 8836	0.274
Creatinine (mg/dL)	1.2 ± 0.7	1.0 ± 0.5	1.1 ± 0.7	1.3 ± 0.8	1.2 ± 0.7	0.005 ^2^
eGFR (mL/min/1.73 m^2^)	75 ± 25	80 ± 23	76 ± 23	72 ± 27	70 ± 24	0.003 ^3^
Glucose (mg/dL)	121 ± 47	121 ± 53	123 ± 50	123 ± 44	115 ± 36	0.534
Hemoglobin (g/dL)	12.5 ± 2.01	12 ± 1.8	12.5 ± 2	13 ± 2.1	12.4 ± 2.2	<0.0014 ^4^
Aspartate aminotransferase (U/L)	32 ± 21	30 ± 19	34 ± 20	31 ± 24	33 ± 22	0.307
Alanine aminotransferase (U/L)	27 ± 24	27 ± 27	27 ± 23	28 ± 24	25 ± 22	0.766
Sodium (mmol/L)	138 ± 6	139 ± 6	138 ± 9	138 ± 4	137 ± 4	0.572
Left ventricular end-diastolic diameter (mm)	48 ± 7	46 ± 6	47 ± 6	49 ± 6	50 ± 7	<0.0015 ^5^
Left ventricular mass (g)	229 ± 78	211 ± 67	216 ± 60	252 ± 83	246 ± 97	<0.0016 ^6^
Relative wall thickness	0.51 ± 0.14	0.52 ± 0.16	0.52 ± 0.12	0.51 ± 0.14	0.48 ± 0.13	0.0257 ^7^
Left atrial diameter (mm)	47 ± 11	43 ± 8	50 ± 11	45 ± 11	53 ± 10	<0.0018 ^8^
Systolic pulmonary artery pressure (mmHg)	52 ± 19	49 ± 17	55 ± 19	49 ± 24	53 ± 17	0.263
Tricuspid annulus plane systolic excursion (mm)	19 ± 4	19 ± 5	18 ± 4	20 ± 4	18 ± 4	0.274
Left ventricular ejection fraction (%)	54 ± 4	54 ± 5	53 ± 4	54 ± 4	53 ± 3	0.039

Superscript numbers correspond to significant post hoc pairwise comparisons (Tukey/Games–Howell) listed below: ^1^ Age: G1 > G3 (*p* = 0.030); G2 > G3 (*p* < 0.001). ^2^ Creatinine: G1 < G3 (*p* = 0.003); G1 < G4 (*p* = 0.034). ^3^ eGFR: G1 > G3 (*p* = 0.035); G1 > G4 (*p* = 0.003). ^4^ Hemoglobin: G1 < G3 (*p* < 0.001); G2 < G3 (*p* < 0.001); G4 < G3 (*p* = 0.001). ^5^ Left ventricular end-diastolic diameter: G1 < G3 (*p* < 0.001); G1 < G4 (*p* < 0.001); G2 < G3 (*p* = 0.002); G2 < G4 (*p* < 0.001). ^6^ Left ventricular mass: G1 < G3 (*p* < 0.001); G1 < G4 (*p* = 0.006); G2 < G3 (*p* < 0.001); G2 < G4 (*p* = 0.021). ^7^ Relative wall thickness: G1 > G4 (*p* = 0.021); G2 > G4 (*p* = 0.033). ^8^ Left atrial diameter: G1 < G2 (*p* < 0.001); G1 < G4 (*p* < 0.001); G2 > G3 (*p* < 0.001); G3 < G4 (*p* < 0.001). Two-way ANOVA models including sex, AF, and their interaction term were performed. No significant sex × AF interactions were observed for any variable. Abbreviations: AF, atrial fibrillation; eGFR, estimated glomerular filtration rate.

**Table 2:** Univariable Cox Proportional Hazards Analysis of Predictors of Heart Failure Rehospitalization in HFpEF.

Variable	*p*-Value	HR (95% CI)
Age	0.017	0.985 [0.973–0.997]
Female sex	0.019	0.705 [0.526–0.945]
Chronic ischemic heart disease	0.410	1.170 [0.805–1.699]
Type 2 diabetes mellitus	0.188	1.221 [0.907–1.644]
Hypertension	0.001	0.571 [0.413–0.791]
Hypercholesterolemia	0.649	0.931 [0.685–1.265]
Smoking	0.834	0.955 [0.620–1.470]
Chronic kidney disease	0.332	1.164 [0.857–1.581]
Atrial fibrillation	0.008	1.490 [1.112–1.998]
NYHA class	<0.001	1.527 [1.222–1.909]
Hemoglobin	0.723	1.010 [0.956–1.067]
Glycemia	0.416	1.001 [0.998–1.004]
Serum sodium	<0.001	0.959 [0.937–0.981]
Aspartate aminotransferase	0.640	0.998 [0.991–1.006]
Alanine aminotransferase	0.199	0.995 [0.987–1.003]
Creatinine	0.996	1.000 [0.808–1.239]
Estimated glomerular filtration rate	0.798	0.999 [0.993–1.005]
Log-transformed NT-proBNP	0.488	1.052 [0.912–1.214]
Left ventricular end-diastolic diameter	0.127	1.017 [0.995–1.040]
Left ventricular mass	0.304	1.001 [0.999–1.003]
Left ventricular relative wall thickness	0.720	0.823 [0.283–2.390]
Left ventricular ejection fraction	0.009	0.949 [0.912–0.987]
Left atrial antero-posterior diameter	<0.001	1.025 [1.014–1.037]
Tricuspid annulus plane systolic excursion	0.019	0.930 [0.875–0.988]
Systolic pulmonary artery pressure	0.020	1.015 [1.002–1.027]
Tricuspid regurgitation more than mild	0.048	1.445 [1.003–2.083]
Mitral regurgitation more than mild	0.012	1.468 [1.089–1.978]
Loop diuretics use	0.073	1.432 [0.967–2.118]
Betablockers use	0.807	1.048 [0.721–1.522]
Angiotensin conversing enzyme inhibitors/Angiotensin receptor neprilysin inhibitors use	0.113	0.786 [0.583–1.059]
Sodium–glucose cotransporter-2 inhibitors use	0.015	1.880 [1.129–3.132]
Mineralocorticoid receptor antagonists use	0.035	1.393 [1.024–1.897]
Antiarrhythmics use	0.408	1.235 [0.749–2.038]

Abbreviations: CI, confidence interval; HR, hazard ratio. Male sex was used as the reference category for the sex variable.

**Table 3:** Multivariable Cox Proportional Hazards Analysis of Predictors of Heart Failure Rehospitalization in HFpEF.

Variable	*p*-Value	HR (95% CI)
Age	0.003	0.981 [0.969–0.994]
Female sex	0.032	0.718 [0.531–0.971]
Atrial fibrillation	0.021	1.449 [1.057–1.987]
NYHA class	0.004	1.414 [1.119–1.788]
Left ventricular ejection fraction	0.036	0.958 [0.920–0.997]
Mitral regurgitation more than mild	0.146	1.267 [0.921–1.742]
Sodium–glucose cotransporter-2 inhibitors use	0.289	1.325 [0.787–2.232]

**Table 4:** Parsimonious multivariable Cox regression model for heart failure rehospitalization in HFpEF.

Variable	Hazard Ratio (HR)	95% CI	*p*-Value
Age (per year)	0.98	0.97–0.99	0.003
Female sex	0.73	0.54–0.99	0.042
Atrial fibrillation	1.51	1.11–2.06	0.008
NYHA class	1.47	1.17–1.85	<0.001
LVEF (%)	0.96	0.92–1.00	0.027

Model performance: C-index = 0.622. Abbreviations: CI, confidence interval; HFpEF, heart failure with preserved ejection fraction; HR, hazard ratio; LVEF, left ventricular ejection fraction; NYHA, New York Heart Association.

**Table 5:** Expanded multivariable Cox regression model for heart failure rehospitalization in HFpEF.

Variable	Hazard Ratio (HR)	95% CI	*p*-Value
Age (per year)	0.98	0.96–0.99	0.002
Female sex	0.75	0.54–1.04	0.081
Atrial fibrillation	1.45	1.05–1.98	0.023
NYHA class	1.38	1.09–1.76	0.008
LVEF (%)	0.95	0.91–0.99	0.017
Estimated glomerular filtration rate	0.99	0.99–1.01	0.438
SGLT2 inhibitor use	1.34	0.79–2.26	0.279
Mineralocorticoid receptor antagonist use	1.13	0.79–1.64	0.503
Loop diuretic use	1.23	0.78–1.94	0.372
LV end-diastolic diameter	1.00	0.97–1.02	0.759

Model performance: C-index = 0.627. Abbreviations: SGLT2, sodium–glucose cotransporter-2. Other abbreviations as in Table 4.

**Table 6:** Four-group sex–AF Cox regression model for heart failure rehospitalization in HFpEF.

Variable	Hazard Ratio (HR)	95% CI	*p*-Value
Age (per year)	0.98	0.96–0.99	0.001
Women with AF	1.13	0.72–1.75	0.599
Men without AF	1.01	0.65–1.58	0.960
Men with AF	1.85	1.18–2.89	0.007
NYHA class	1.38	1.08–1.75	0.009
LVEF (%)	0.95	0.91–0.99	0.012
Estimated glomerular filtration rate	0.99	0.99–1.01	0.399
SGLT2 inhibitor use	1.32	0.79–2.22	0.294
Mineralocorticoid receptor antagonist use	1.14	0.80–1.64	0.468
Loop diuretic use	1.25	0.79–1.97	0.347

Model performance: C-index = 0.634. Reference group: Women without AF. Abbreviations as in Table 4 and Table 5.

## Data Availability

The raw data supporting the conclusions of this article will be made available by the authors upon reasonable request and following approval by the University of Medicine and Pharmacy of Craiova, Romania. The data presented in this study are not publicly available due to privacy and ethical restrictions.

## Abbreviations

The following abbreviations are used in this manuscript:

AF	Atrial fibrillation
ANOVA	Analysis of variance
CI	Confidence interval
eGFR	Estimated glomerular filtration rate
HF	Heart failure
HFpEF	Heart failure with preserved ejection fraction
HR	Hazard ratio
LV	Left ventricular
LVEF	Left ventricular ejection fraction
NT-proBNP	N-terminal pro-B-type natriuretic peptide
NYHA	New York Heart Association
SGLT2i	Sodium–glucose cotransporter-2 inhibitor
TAPSE	Tricuspid annulus plane systolic excursion

## References

[1] Bidaoui G., Assaf A., Marrouche N. (2025). Atrial Fibrillation in Heart Failure: Novel Insights, Challenges, and Treatment Opportunities. Curr. Heart Fail. Rep..

[2] Reddy Y.N.V., Borlaug B.A., Gersh B.J. (2022). Management of Atrial Fibrillation Across the Spectrum of Heart Failure with Preserved and Reduced Ejection Fraction. Circulation.

[3] McDonagh T.A., Metra M., Adamo M., Baumbach A., Böhm M., Burri H., Čelutkiene J., Chioncel O., Cleland J.G.F., Coats A.J.S. (2021). 2021 ESC Guidelines for the Diagnosis and Treatment of Acute and Chronic Heart Failure. Eur. Heart J..

[4] Espnes H., Wilsgaard T., Ball J., Løchen M.-L., Njølstad I., Schnabel R.B., Gerdts E., Sharashova E. (2025). Heart Failure in Atrial Fibrillation Subtypes in Women and Men in the Tromsø Study. JACC Adv..

[5] Andrade J.G., Deyell M.W., Lee A.Y.K., Macle L. (2018). Sex Differences in Atrial Fibrillation. Can. J. Cardiol..

[6] Goette A., Corradi D., Dobrev D., Aguinaga L., Cabrera J.-A., Chugh S.S., De Groot J.R., Soulat-Dufour L., Fenelon G., Hatem S.N. (2024). Atrial Cardiomyopathy Revisited—Evolution of a Concept: A Clinical Consensus Statement of the European Heart Rhythm Association (EHRA) of the ESC, the Heart Rhythm Society (HRS), the Asian Pacific Heart Rhythm Society (APHRS), and the Latin American Heart Rhythm Society (LAHRS). Europace.

[7] Winters J., Isaacs A., Zeemering S., Kawczynski M., Maesen B., Maessen J., Bidar E., Boukens B., Hermans B., Van Hunnik A. (2023). Heart Failure, Female Sex, and Atrial Fibrillation Are the Main Drivers of Human Atrial Cardiomyopathy: Results From the CATCH ME Consortium. J. Am. Heart Assoc..

[8] Gorczyca-Głowacka I., Molenda K., Nadel M., Galas A., Tymińska A., Byczkowska K., Siniarski A., Furmanek W., Stefański A., Wożakowska-Kapłon B. (2025). Integrated Care in Patients with Atrial Fibrillation and Optimal Medical Treatment for Heart Failure: Results from the Heart failuRe ObsErvational Study (HEROES). J. Clin. Med..

[9] Van Gelder I.C., Rienstra M., Bunting K.V., Casado-Arroyo R., Caso V., Crijns H.J.G.M., De Potter T.J.R., Dwight J., Guasti L., Hanke T. (2024). 2024 ESC Guidelines for the Management of Atrial Fibrillation Developed in Collaboration with the European Association for Cardio-Thoracic Surgery (EACTS). Eur. Heart J..

[10] Galderisi M., Cosyns B., Edvardsen T., Cardim N., Delgado V., Di Salvo G., Donal E., Sade L.E., Ernande L., Garbi M. (2017). Standardization of Adult Transthoracic Echocardiography Reporting in Agreement with Recent Chamber Quantification, Diastolic Function, and Heart Valve Disease Recommendations: An Expert Consensus Document of the European Association of Cardiovascular Imaging. Eur. Heart J. Cardiovasc. Imaging.

[11] Lang R.M., Badano L.P., Mor-Avi V., Afilalo J., Armstrong A., Ernande L., Flachskampf F.A., Foster E., Goldstein S.A., Kuznetsova T. (2015). Recommendations for Cardiac Chamber Quantification by Echocardiography in Adults: An Update from the American Society of Echocardiography and the European Association of Cardiovascular Imaging. Eur. Heart J. Cardiovasc. Imaging.

[12] Barillas-Lara M.I., Monahan K., Helm R.H., Vasan R.S., Schou M., Køber L., Gislason G., Torp-Pedersen C., Andersson C. (2021). Sex-Specific Prevalence, Incidence, and Mortality Associated with Atrial Fibrillation in Heart Failure. JACC Clin. Electrophysiol..

[13] Emdin C.A., Wong C.X., Hsiao A.J., Altman D.G., Peters S.A., Woodward M., Odutayo A.A. (2016). Atrial Fibrillation as Risk Factor for Cardiovascular Disease and Death in Women Compared with Men: Systematic Review and Meta-Analysis of Cohort Studies. BMJ.

[14] Feinberg J.B., Nielsen E.E., Kjeldsen S.E., Devereux R.B., Gerdts E., Wachtell K., Olsen M.H. (2023). Sex Differences in Atrial Fibrillation and Associated Complications in Hypertensive Patients with Left Ventricular Hypertrophy: The LIFE Study. Am. J. Hypertens..

[15] Odening K.E., Deiß S., Dilling-Boer D., Didenko M., Eriksson U., Nedios S., Ng F.S., Roca Luque I., Sanchez Borque P., Vernooy K. (2019). Mechanisms of Sex Differences in Atrial Fibrillation: Role of Hormones and Differences in Electrophysiology, Structure, Function, and Remodelling. EP Eur..

[16] Dewan P., Rørth R., Raparelli V., Campbell R.T., Shen L., Jhund P.S., Petrie M.C., Anand I.S., Carson P.E., Desai A.S. (2019). Sex-Related Differences in Heart Failure with Preserved Ejection Fraction. Circ. Heart Fail..

[17] Preda A., Giordano F., Giani V., Guarracini F., Mazzone P. (2023). Accelerated Adverse Atrial Remodeling in Women with Atrial Fibrillation: Results from Studies Using Electroanatomic Mapping Systems. Am. J. Cardiol..

[18] Iovănescu M.L., Hădăreanu D.R., Toader D.M., Florescu C., Istrătoaie O., Donoiu I., Militaru C. (2023). The Impact of Atrial Fibrillation on All Heart Chambers Remodeling and Function in Patients with Dilated Cardiomyopathy—A Two- and Three-Dimensional Echocardiography Study. Life.

[19] Bergau L., Bengel P., Sciacca V., Fink T., Sohns C., Sommer P. (2022). Atrial Fibrillation and Heart Failure. J. Clin. Med..

[20] Son Y.-J., Kim D.-Y., Won M.H. (2021). Sex Differences in the Association between Atrial Fibrillation and 90-Day Adverse Outcomes among Older Adults with Heart Failure: A Retrospective Cohort Study. Int. J. Environ. Res. Public Health.

[21] Florescu D.R., Muraru D., Florescu C., Volpato V., Caravita S., Perger E., Bălșeanu T.A., Parati G., Badano L.P. (2021). Right Heart Chambers Geometry and Function in Patients with the Atrial and the Ventricular Phenotypes of Functional Tricuspid Regurgitation. Eur. Heart J.—Cardiovasc. Imaging.

[22] Hădăreanu C.-D., Hădăreanu D.-R., Stoiculescu F.-M., Raicea V.-C., Târtea G.-C., Florescu C., Radu R.I., Donoiu I. (2024). The Added Value of Advanced Echocardiography for the Morpho-Functional and Prognostic Evaluation of the Right Heart in Dilated Cardiomyopathy: Do Not Forget about the Right Atrium. J. Clin. Med..

[23] Tomaselli M., Penso M., Badano L.P., Radu N., Springhetti P., Buta A., Benzoni G., Hădăreanu D.R., Caravita S., Baratto C. (2026). Sex-Specific Differences in Right Heart Remodelling and Patient Outcomes in Secondary Tricuspid Regurgitation. Eur. Heart J.—Cardiovasc. Imaging.

[24] Sanna G.D., Erre G.L., Cameli M., Guerra F., Pastore M.C., Marini A., Campora A., Gironella P., Costamagna M., Mandoli G.E. (2024). Association of Sex with In-Hospital Management and Outcomes of Patients with Heart Failure: Data from the REAL-HF Registry. Am. Heart J..

[25] Stoiculescu F.-M., Hădăreanu D.-R., Hădăreanu C.-D., Donoiu I., Istrătoaie O., Raicea V.-C., Florescu C. (2025). Prediction Model of Rehospitalization and Mortality in Heart Failure Patients with Preserved and Mildly Reduced Ejection Fraction: The AD2NNER Risk Score. Front. Cardiovasc. Med..

[26] Solomon S.D., McMurray J.J.V., Claggett B., de Boer R.A., DeMets D., Hernandez A.F., Inzucchi S.E., Kosiborod M.N., Lam C.S.P., Martinez F. (2022). Dapagliflozin in Heart Failure with Mildly Reduced or Preserved Ejection Fraction. N. Engl. J. Med..

[27] Anker S.D., Butler J., Filippatos G., Ferreira J.P., Bocchi E., Böhm M., Rocca H.-P.B., Choi D.-J., Chopra V., Chuquiure-Valenzuela E. (2021). Empagliflozin in Heart Failure with a Preserved Ejection Fraction. N. Engl. J. Med..

[28] McDonagh T.A., Metra M., Adamo M., Gardner R.S., Baumbach A., Böhm M., Burri H., Butler J., Čelutkienė J., Chioncel O. (2023). 2023 Focused Update of the 2021 ESC Guidelines for the Diagnosis and Treatment of Acute and Chronic Heart Failure: Developed by the Task Force for the Diagnosis and Treatment of Acute and Chronic Heart Failure of the European Society of Cardiology (ESC) with the Special Contribution of the Heart Failure Association (HFA) of the ESC. Eur. Heart J..

[29] Noubiap J.J., Pathak R.K., Thomas G., Elliott A.D., Sanders P., Middeldorp M.E. (2024). Sex Differences in Outcomes of an Intensive Risk Factor Modification Program in Patients with Atrial Fibrillation. Circ. Arrhythmia Electrophysiol..

[30] Rillig A., Magnussen C., Ozga A.-K., Suling A., Brandes A., Breithardt G., Camm A.J., Crijns H.J.G.M., Eckardt L., Elvan A. (2021). Early Rhythm Control Therapy in Patients with Atrial Fibrillation and Heart Failure. Circulation.

[31] Kirchhof P., Camm A.J., Goette A., Brandes A., Eckardt L., Elvan A., Fetsch T., van Gelder I.C., Haase D., Haegeli L.M. (2020). Early Rhythm-Control Therapy in Patients with Atrial Fibrillation. N. Engl. J. Med..

[32] Kelly J.P., DeVore A.D., Wu J., Hammill B.G., Sharma A., Cooper L.B., Felker G.M., Piccini J.P., Allen L.A., Heidenreich P.A. (2019). Rhythm Control Versus Rate Control in Patients with Atrial Fibrillation and Heart Failure with Preserved Ejection Fraction: Insights from Get with the Guidelines—Heart Failure. J. Am. Heart Assoc..

[33] Chen S., Pürerfellner H., Meyer C., Acou W.-J., Schratter A., Ling Z., Liu S., Yin Y., Martinek M., Kiuchi M.G. (2020). Rhythm Control for Patients with Atrial Fibrillation Complicated with Heart Failure in the Contemporary Era of Catheter Ablation: A Stratified Pooled Analysis of Randomized Data. Eur. Heart J..

[34] Stoiculescu F.-M., Hădăreanu D.-R., Hădăreanu C.-D., Iovănescu M.-L., Târtea G.-C., Donoiu I., Cojocaru P.-A., Militaru S., Istrătoaie O., Florescu C. (2026). A Four-Phenotype Model for Risk Stratification in Heart Failure with Preserved and Mildly Reduced Ejection Fraction: The Role of Sex and Diabetes. Biomedicines.

[35] Hahn R.T., Lindenfeld J., Böhm M., Edelmann F., Lund L.H., Lurz P., Metra M., Tedford R.J., Butler J., Borlaug B.A. (2024). Tricuspid Regurgitation in Patients with Heart Failure and Preserved Ejection Fraction. J. Am. Coll. Cardiol..

[36] The Task Force for the Management of COVID-19 of the European Society of Cardiology (2022). ESC Guidance for the Diagnosis and Management of Cardiovascular Disease during the COVID-19 Pandemic: Part 2—Care Pathways, Treatment, and Follow-Up. Eur. Heart J..

